# CamOptimus: a tool for exploiting complex adaptive evolution to optimize experiments and processes in biotechnology

**DOI:** 10.1099/mic.0.000477

**Published:** 2017-06-21

**Authors:** Ayca Cankorur-Cetinkaya, Joao M. L. Dias, Jana Kludas, Nigel K. H. Slater, Juho Rousu, Stephen G. Oliver, Duygu Dikicioglu

**Affiliations:** ^1^​ Cambridge Systems Biology Centre and Department of Biochemistry, University of Cambridge, Cambridge, CB2 1GA, UK; ^2^​ Wellcome Trust Sanger Institute, Wellcome Trust Genome Campus, Hinxton, Cambridge, CB10 1SA, UK; ^3^​ Department of Haematology, Cambridge University Hospitals NHS Trust, Cambridge, CB2 0QQ, UK; ^4^​ Helsinki Institute for Information Technology HIIT; Department of Computer Science, Aalto University, Konemiehentie 2, Espoo, FI-02150, Finland; ^5^​ Department of Chemical Engineering and Biotechnology, University of Cambridge, Cambridge CB3 0AS, UK; ^†^​ Present address: Department of Chemical Engineering and Biotechnology, University of Cambridge, Cambridge CB3 0AS, UK.

**Keywords:** experimental design tool, evolutionary algorithms, genetic algorithm, symbolic regression, recombinant protein production, *Pichia pastoris*

## Abstract

Multiple interacting factors affect the performance of engineered biological systems in synthetic biology projects. The complexity of these biological systems means that experimental design should often be treated as a multiparametric optimization problem. However, the available methodologies are either impractical, due to a combinatorial explosion in the number of experiments to be performed, or are inaccessible to most experimentalists due to the lack of publicly available, user-friendly software. Although evolutionary algorithms may be employed as alternative approaches to optimize experimental design, the lack of simple-to-use software again restricts their use to specialist practitioners. In addition, the lack of subsidiary approaches to further investigate critical factors and their interactions prevents the full analysis and exploitation of the biotechnological system. We have addressed these problems and, here, provide a simple‐to‐use and freely available graphical user interface to empower a broad range of experimental biologists to employ complex evolutionary algorithms to optimize their experimental designs. Our approach exploits a Genetic Algorithm to discover the subspace containing the optimal combination of parameters, and Symbolic Regression to construct a model to evaluate the sensitivity of the experiment to each parameter under investigation. We demonstrate the utility of this method using an example in which the culture conditions for the microbial production of a bioactive human protein are optimized. CamOptimus is available through: (https://doi.org/10.17863/CAM.10257).

## Introduction

Advances in synthetic biology facilitate the rapid and efficient engineering of microbial cell factories for the production of novel chemical compounds and recombinant proteins [1]. Synthetic biology approaches allow multi-level modification of the host organisms through the development of synthetic promoters, codon-optimized synthetic reading-frames, artificial transcription factors, and insertion of expression cassettes, to improve the quality and yield of the target product [2]. The success of such modifications needs to be evaluated under environmental conditions that are suitable for the desired application, since the performance of the constructed strain is usually highly condition-dependent [3]. Thus, the optimization of the environmental conditions is important, not only for large-scale production in biotechnology, but also for laboratory-scale screening and testing of potential high-performing strains.

While Design of Experiments (DOE) is a commonly used method for the optimization of such systems, and is suitable for addressing low-dimensional design problems, it is impractical for optimization problems that require the investigation of a large number of factors at many different levels and modelling their output response. This is due to the so-called explosion problem in Factorial Design that is caused by an exponential increase in the number of experiments required as the number of conditions or levels to be tested increases. This usually leads to the artificial limitation of the number of factors, and their levels, to be examined. While this reduction introduces the risk of overlooking some critical components of design, only this can permit simple regression techniques, available in most DOE tools, to be used to model the data. However, Yang *et al.* [4] report that these regression techniques frequently fail in their estimations of high-dimensional datasets. In spite of this, the availability of user-friendly computational tools encourages the utilization of DOE even for highly multifactorial problems, by prioritizing the factors and reducing the dimensionality of the problem. Apart from the obvious limitations of the DOE approach, many potential users may be constrained by the fact that there do not appear to be any user-friendly DOE tools that are publicly accessible.

These issues concerning the DOE have led to the exploration of the utility of non-statistical approaches, such as Artificial Intelligence (AI), as tools for solving such multiparameter optimization problems [5]. Among these approaches, Genetic Algorithms (GAs) were shown to be well suited for the optimization of a wide range of practical problems in science, engineering and industry [6]. The GA methodology borrows concepts such as population, generation, gene, chromosome, mutation, cross-over, and mating from Evolutionary Genetics, and redefines them in a computational framework that mimics Natural Selection. The search starts with a set of experimental conditions to be tested, which is comprised of the factors of interest at their randomly assigned levels from within a predetermined allowable range. A new set of experimental conditions to be tested is generated based on the success of the previous set and, over time, the search is guided towards a more confined space, where the global optimum resides, depending on how different sets of conditions perform in achieving the desired objective.

GA has the advantage of exploring a large variable space without exponentially increasing the number of experiments that need to be conducted, and it only requires the maximum and minimum values for any number of factors to be known in order to define the boundaries of the possible solution space [6]. In contrast to fractional factorial design, with GA, the number of factors and the number of levels at which each of these factors is investigated does not result in an explosion problem. Genetic algorithms have been applied in different areas of biological research ranging from synthetic biology and metabolic engineering applications [7–9], to the optimization of the design of an *in situ* bioremediation system [10], or to the prediction of RNA secondary structure [11], or to multiple sequence alignment [12, 13]. Despite their advantages over DOE when multiple parameters at a large number of levels need to be optimized, GAs have been neglected by the research community. This is a consequence of the lack of availability of simple-to-use computational tools for conducting the optimization, and the dearth of accompanying approaches to model and investigate the response.

Here, we describe a hybrid methodology that employs genetic programming as its working principle, to address the multi-parameter experimental design problem. We present a platform, CamOptimus (https://doi.org/10.17863/CAM.10257), to overcome the concerns and limitations associated with the various platforms available (discussed above). We demonstrate the applicability of our approach and its success on a biotechnology application – recombinant protein production by the industrial yeast, *Komagataella phaffii* (formerly *Pichia pastoris*). The environmental conditions used to cultivate the Human Lysozyme (HuLy)-producing cells under inducible AOX (alcohol oxidase) promoter were optimized to increase the protein titres, and up to 80 % improvement was achieved in comparison to the previously reported conditions. The software allows the user to: (i) collect extensive data over the complete experimental space of interest and identify the optimal design within that search space by employing a GA, and (ii) describe this space by constructing an evolutionary model using Symbolic Regression (SR) and identify the critical factors for the system under investigation. A detailed user manual, the source codes, and the CamOptimus platform (for both Windows OS and Mac OSX) are available under free licensing (GNU General Public License v3.0) at https://doi.org/10.17863/CAM.10257.

## Methods

### Strain and growth conditions

Haploid *K.* *phaffii* strain GS115, expressing Human Lysozyme protein under the control of the methanol-inducible *AOX1* promoter [14, 15], was employed in the study. Pre-cultures were prepared in YPG [composed of 1 % (w/v) yeast extract, 2 % (w/v) peptone and 2 % (w/v) glycerol], with a single colony selected from YPAG plates, which contain 2 % (w/v) agar in addition to YPG composition. The cultures (3 ml) were inoculated from the pre-culture grown overnight (OD_600_≅5.00). Experiments were carried out in 14 ml round-bottom, dual snap-cap polypropylene tubes (BD Falcon). Media formulations were prepared using the same stock solutions of each medium component throughout the experiment to eliminate variability over ‘generations’. The initial pH of each individual medium composition was measured in dummy cultures and the tuning of the pH to set it at its assigned level was determined using undiluted HCl (37 %) or NaOH solution (1M). The pH was maintained constant using citrate-phosphate buffer, which has an allowable working range of pH 2.6–7.0. Cells were cultivated at 30 °C at an agitation rate of 200 r.p.m. The optical density at 600 nm was monitored as a proxy for biomass concentration and was measured prior to induction and during harvest. A 1 ml sample of the culture was harvested by centrifugation at 3000***g*** for 10 min at 4 °C, and stored at −20 °C until further analysis.

### Analytical methods

The activity of the secreted enzyme was determined using EnzChek Lysozyme Assay Kit (Molecular Probes) as described by the manufacturer [16]. The glycerol content was determined enzymatically using UV-based methods as described by the manufacturer (Glycerol Kit; r-biopharm) [17].

### Determination of culture induction and harvest times

The optimal induction and harvest periods were determined prior to the optimization experiments. For this purpose, cells were grown in unbaffled shake flasks using previously reported values for the medium compositions and initial pH of the culture [18]. The glycerol remaining in the cultures was determined during the 8 h period of 24–32 h post-inoculation. The induction time was selected as 30 h post-inoculation in order to ensure at least 6 h of glycerol starvation for the culture. The activity of the secreted HuLy was determined during the period of 30–60 h post-induction. The harvest time was selected as 42 h to ensure no further increase in enzyme activity but to allow sufficient time for maximal utilization of resources.

### Experimental set-up for the optimization study

A total of nine cultivation parameters: (NH_4_)_2_HPO_4_, KCl, MgSO_4_ . 7H_2_O, FeSO_4_ . 7H_2_O, CaCl_2_ . 2H_2_O, glycerol, methanol, sorbitol and pH, were selected to be optimized and the range within which the levels of each parameter could lie was determined from the literature [15, 19–22]. Four objectives were selected for the optimization study: maximization of biomass production during the growth-induced pre-induction phase, minimization of biomass production throughout the induction phase, maximization of the total secreted HuLy activity, and maximization of the specific productivity [defined as the total lysozyme activity per unit biomass (OD_600_)]. Experiments were carried out in triplicate to allow for the variability within each experimental condition.

### GA methodology and parameter selection

The GA methodology was implemented as described by Sarma *et al.* [8]. The terminology employed in the methodology is adopted from Evolutionary Genetics; throughout the text, the terminology provided in quotation marks follows the definitions provided in Table 1.

**Table 1.** GA – experimental protocol conversion table for commonly employed terminology

GA term	Equivalent in the current experimental setup
Gene	Individual experimental factor
Number of bits (b)	Number of binary digits (0 or 1) assigned to describe the value (i.e. the ‘length’) of each ‘gene’
2^b^	Number of levels to which each ‘gene’ can be assigned
Chromosome	Individual set of conditions to be tested experimentally
Generation	Each round of experiments
Population	Number of ‘chromosomes’ to be experimentally tested in each ‘generation’
Evolution	Narrowing down the range of experimental conditions to reach the required objective through a course of consecutive rounds of experiments (‘generations’)
Score	Evaluation of how well suited the condition is to achieving the required objective (how ‘fit’ the ‘chromosome’ is)
Parent	One of the two ‘chromosomes’ to undergo genetic hybridization
Child/Offspring	One of the two new ‘chromosomes’ generated
Mating	Process by which two ‘parent chromosomes’ recombine to yield the two ‘children’
Cross-over	Point where the recombination event occurs
Mutation	A random change in the bit value (0 to 1 or 1 to 0) introduced with an assigned probability

The nine factors were selected to be of 5 ‘bit’ length each and 2^5^=32 levels were assigned to each of these factors within their allowable range. Each ‘chromosome’ comprised of 9 ‘5-bit’ long ‘genes’, yielding a total length of 45 ‘bits’. The ‘population’ size was set to 16 individuals (2×N−2). The normalized objective function used to evaluate the ‘fitness score’ was:

(1) $$\begin{array}{ll} Obj=\;\;\;\; & maximize\{w1\times \frac{OD_{bi}-{\left(OD_{bi}\right)}_{min_{i}}}{{\left(OD_{bi}\right)}_{max_{i}}-{\left(OD_{bi}\right)}_{min_{i}}}\\ & +w2\times (1-\frac{\left[\left(OD_{ai}-OD_{bi}\right)-{\left(OD_{ai}-OD_{bi}\right)}_{min_{i}}\right]}{\left[{\left(OD_{ai}-OD_{bi}\right)}_{max_{i}}-{\left(OD_{ai}-OD_{bi}\right)}_{min_{i}}\right]})\\ & +w3\times \frac{Ea-{\left(Ea\right)}_{min_{i}}}{{\left(Ea\right)}_{max_{i}}-{\left(Ea\right)}_{min_{i}}}+w4\times \frac{P-{\left(P\right)}_{min_{i}}}{{\left(P\right)}_{max_{i}}-{\left(P\right)}_{min_{i}}}\} \end{array}$$

where OD_bi_ = cell culture OD before induction

OD_ai_ = cell culture OD at harvest

Ea = HuLy activity

P = specific productivity

(OD_bi_)min_i_ = Minimum of the OD before induction over generation i

(OD_ai_−OD_bi_)min_i_ = Minimum of the OD difference between after induction and before induction over generation i

(Ea)min_i_ = Minimum of the HuLy activity over generation i

(P)min_i_ = Minimum of the specific productivity over generation i

(OD_bi_)max_i_ = Maximum of the OD before induction over generation i

(OD_ai_−OD_bi_)max_i_ = Maximum of the OD difference between after induction and before induction over generation i

(Ea)max_i_ = Maximum of the HuLy activity over generation i

(P)max_i_ = Maximum of the specific productivity over generation i

w1=w2=w3=w4=0.25

The median of the replicate values was used to determine the fitness of each ‘chromosome’. Single missing values in the experiments were replaced by the arithmetic average of the remaining two replicates. The Roulette-wheel selection method, which allows ‘fitter’ individuals to have more chance to be selected as parents [23], was used with a selection probability of 0.5 for the identification of the ‘fitter’ individuals within the ‘population’ to ‘mate’. Both ‘cross-overs’ and ‘mutations’ were introduced at single locations on the ‘chromosome’ in order to introduce ‘genetic’ variability in the next ‘generation’. The ‘mutation’ rate and the ‘cross-over’ rate were selected as 0.1 and 0.9 per ‘generation’, respectively. New ‘generations’ of experiments were carried out until the average ‘fitness’ of the ‘population’, as indicated by the enzyme activity and specific productivity, displayed convergence.

### Evaluation of the convergence behaviour and fine-tuning of the optimum

The convergence of the levels of individual factors over the course of ‘generations’ was investigated employing ‘population’ profiling analysis [8] with slight modifications. The absolute frequency of each individual level of every factor was determined for the three ‘generations’ to monitor the levels around which convergence was observed throughout ‘evolution’. A % occupancy was defined as the relative representation of each level employed in the better-performing fraction (i.e. the half of the ‘population’ with the highest overall score in the Test Case) of the last ‘generation’ provided as a percent fraction. This information was used to create a footprint for each factor and was used in combination with population profiling to determine the optimal level of each factor.

### Regression analysis

Microsoft Excel Analysis ToolPak Add-in was used to conduct multiple linear regression (MLR). GPTIPS2, a symbolic regression platform, was employed for creating evolutionary models [24, 25]. The default settings of the software were adopted, except for the ‘population’ size and the number of ‘generations’, both of which were set to 500. The allowable mathematical operations were selected as addition, subtraction, multiplication and division. The platform was managed in the MATLAB (v8.2.0.701) environment.

### Executable stand-alone with the Graphical User Interface (GUI)

The executable compilation and the stand-alone version of the software were developed using MATLAB release 9.0.0.341360 and the MATLAB Runtime environment release 9.0.1. The stand-alone user interface is available for both Microsoft Windows OS and MAC OS X operating systems. The default parameters in the user interface were taken as described earlier, except for the ‘mutation’ rate in GA analysis, whose default value was set to 0.01. The files for the user manual, the executable and the standalone versions can be accessed from (https://doi.org/10.17863/CAM.10257).

## Results and Discussion

We adopted a two-stage strategy that employs evolutionary algorithms for handling multi-parametric optimization problems in biological systems. The optimization pipeline starts by using the GA to investigate the possible space within which the solution lies. This then leads to the definition of a more restricted space that contains an optimal solution. Finally, in the second stage, SR is employed to obtain a model that will allow the response of the system to incremental changes in the levels of the different factors to be calculated. We adopted this pipeline to investigate how the levels of the environmental cultivation parameters could be optimized to achieve improved yields of HuLy produced by a genetically engineered strain of the industrial yeast, *K. phaffii*. Finally, we developed a simple Graphical User Interface (GUI) for this tool and have made it available to the wider research community.

### GA as a design and optimization tool

The application of GA to biological systems operates as a multi-stage decision-making process, which requires: (i) the determination of the biological objective(s); (ii) the identification of the factors affecting this set of objective(s); (iii) the designation of the allowable range of values/levels for each factor; and (iv) the construction of the objective (‘fitness’) function. Once the structure of the methodology is established, the approach is then used to initially perform experiments for a randomly generated ‘population’, and then to evaluate the ‘fitness’ of each individual within that ‘population’ based on the defined objective function. This evaluation leads to the identification of a new ‘population’ by applying ‘selection’, ‘cross-over’ and ‘mutation’ operators in a similar manner to our understanding of biological evolution, based on the fitness of individuals in the previous generation. The search comprises the creation, testing and selection of new ‘generations’ of ‘populations’ within the search space and is pursued until a satisfactory outcome, described as the termination criterion, is achieved.

We employed the GA methodology to optimize a set of environmental parameters, which we identified to be important for the cultivation of *K. phaffii* expressing HuLy under the inducible *AOX* promoter. *K. phaffii* is widely employed for recombinant protein production due to the high product titres obtained with this host organism, which can be grown at very high cell densities [26]. A number of studies have focused on improving the productivity of this host organism by either designing new expression vectors, optimizing the copy number of the gene encoding the recombinant protein, or by engineering the glycosylation and secretory pathways. The reported cultivation conditions and medium compositions used in these studies display a huge variation depending on the recombinant protein that was produced, the promoter from which the transgene was expressed, or the fermentation mode employed (batch, fed-batch, or continuous) [15, 18, 21, 27]. This model system allowed us to test the effect of many interdependent parameters on a multitude of biological objectives that required optimization (including those that compete with one another). It thus served as a suitable system to demonstrate the flexibility of our approach and evaluate its performance. Moreover, understanding the effects of different parameters and their interactions on the productivity of this expression system would provide its users with valuable knowledge with which to control the processes associated with its cultivation and production.

The first stage of the decision-making process is the determination of the biological objective. For this system, the main goal was to improve the yield of biologically active recombinant protein. For this purpose, four main biological objectives were selected for our system (Supplementary Material): (i) maximizing cell density prior to induction of recombinant protein synthesis; (ii) maximizing the biological activity of the recombinant enzyme at the end of the fermentation; (iii) maximizing the culture’s specific productivity; and (iv) minimizing any further increase in cell density during the induction phase.

An objective function was then constructed to incorporate these different biological objectives so that it could serve as a proxy for ‘fitness’, which was to be calculated from experimental measurements. The objective function was assembled as the weighted sum of the normalized output responses representing the biological objectives of the study. In this test case, we assigned equal weights to each of these biological objectives since there was no evidence to suggest that one or more of these objectives would make a more substantial contribution to the ‘fitness’ score than any of the others.

Factors that may affect biomass and recombinant protein production were identified as the parameters, and the relationships between these selected parameters were taken into consideration. In the present study, we employed *a priori* knowledge of the system by mining the available literature and conducting some preliminary experiments in order to identify the factors to be investigated. Eight medium parameters (ammonium, potassium, magnesium, calcium, iron, glycerol, methanol and sorbitol) and one environmental parameter (pH) were identified as being important to optimize (Supplementary Material). It would be imperative to avoid limiting the number of factors to be investigated in the absence of such *a priori* knowledge. Once the factors were determined, the range of values each parameter would be allowed to assume was set, thus defining the boundaries of the search space. The range was kept as broad as possible, taking into consideration aspects such as toxicity, feasibility or background knowledge, in order to reduce the risk of missing the optimum.

GA requires the determination of certain parameters intrinsic to the concept of evolution implemented within the programming scheme. These parameters needed to be specified initially and kept constant throughout the procedure. One of these parameters is the size of the ‘population’, which would be investigated in each ‘generation’ of experiments. We established a linear relationship between the population size and the number of factors under investigation. Although there is no theoretical limit to the number of individuals allowed, earlier reports suggested that the optimal ‘population’ size should be confined to the range between n and 2n, where n is the number of ‘genes’, i.e. the factors under investigation [28]. We fixed the size of the ‘population’ to the following relationship, 2×n−2, to remain within the upper quantile of this recommended limit. Consequently, the size of the ‘population’ was 16 for our case study, where we investigated nine factors.

An overcrowded ‘population’ would delay the convergence of the parameters towards an optimum, whereas the search would risk rapidly hitting a local optimum in a small and confined ‘population’ [29]. While with GA, in contrast to DOE, the number of experiments required does not show an exponential increase with the number of parameters investigated, it should be remembered that the population size still increases linearly with the number of factors and consideration needs to be given to the resource implications, in terms of money, personnel and equipment, that a given population size implies.

The final decision to be made regarding the ‘populations’ is the number of levels at which each factor would be tested. In the present study, we allowed each factor to be investigated at 32 different levels within their assigned ranges. There is no theoretical limit to the number of levels that could be investigated, and introducing more levels would naturally improve the precision of the optimum achieved. However, consideration should be given to the degree of precision that is necessary, useful or, indeed, achievable using the techniques employed in the experiments. Adopting the population characteristics discussed above allowed us to investigate the interactive effect of nine environmental parameters, each at 32 different possible levels. A similar experimental regime employing the full factorial DOE would have been equivalent to conducting 32^9^ experiments, that number being equivalent to nearly as many cells as there are in the human body. This comparison demonstrates that GA is a much more rapid and less costly alternative to the solution of multi-parameter optimization problems suffering from high dimensionality.

GA employs ‘survival of the fittest’ as its core working principle to find the optimal solution to a problem [30]. The search was initiated with a randomly created ‘population’. For our test case, the initial randomly created ‘population’ comprised 16 different conditions. The cells were cultivated in these 16 different medium compositions with changing levels of six medium components and pH values, and were induced with addition of different levels of methanol and sorbitol following a 30 h pre-induction phase. The final samples were collected 42 h later than the induction to determine cell density and protein levels. The ‘fitness’ of each individual was then evaluated by calculating the score output of the objective function as defined earlier, and a new ‘population’ was generated as described in Methods. The operations employed in the generation of a new ‘population’ require some parameters to be set. The default values for the selection probability, ‘cross-over rate’ and ‘mutation’ probability were set as 0.5, 0.9 and 0.01, respectively, based on the recommendations in previous reports [30] in order to make the algorithm applicable for a wide range of optimization problems. However, the suitability of these values depends on the problem under investigation and specific applications may require adjustments to these default values. A ‘mutation’ probability of 0.01 was much lower than that employed in earlier studies on the application of GA for medium optimization for biological systems. A ‘mutation’ rate of 0.15 was previously shown to be suitable for the optimization of the medium conditions for microbial cells [8]. Therefore, we adjusted the mutation rate and set it to 0.1 in the case study, in line with earlier reports, although much lower mutation rates were recommended for GA applications in general.

**Fig. 1.**
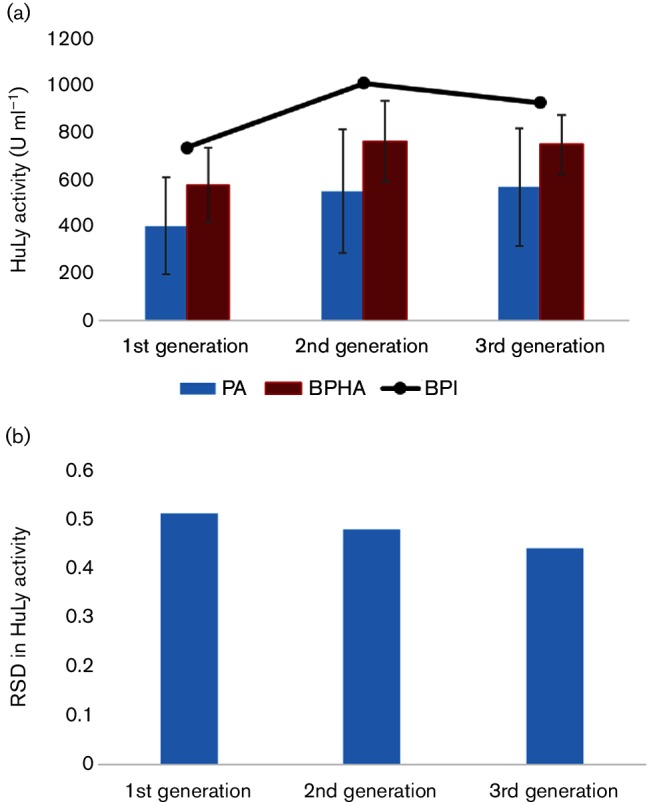
Metrics of the convergence score in finding the optimized sub-space of environmental parameters for improving recombinant production yield. The average enzyme activity scores for all the individuals in the ‘population’ are displayed in blue for each generation represented in the abscissa (PA). The average scores for the better-performing fraction (50 %) of the ‘population’ are displayed similarly in red (BPHA). The error bars represent the variation among the individuals in each ‘population’. The black solid line connecting the black markers represents the trajectory of the best-performing individual (BPI) in each ‘generation’ (a). The contraction of the optimal sub-space through the course of this heuristic search was represented by the relative standard deviation (RSD) in the better-performing fraction of each ‘generation’ (b).

The GA search was conducted for three generations to optimize cultivation conditions for improving HuLy production yield (Supplementary Material). Different criteria could be selected to terminate the execution of the GA. We monitored the average individual objectives scores in each ‘generation’ and terminated the study when there was no further improvement between the average enzyme activity scores, between successive ‘generations’ (these proved to be the second and the third ‘generations’). There was a significant (*P*<0.05) improvement in scores for the better-performing fraction of the ‘population’ between the first and the second ‘generations’, but no significant improvement in scores (*P*>0.1) between the second and the third ‘generations’ (Fig. 1a). The relative standard deviation (RSD), calculated as the standard deviation of the enzyme activity scores normalized by the average enzyme activity score, was used to investigate how the search space was occupied in consecutive ‘generations’. A relative decrease in the RSD value was observed in the better-performing fraction of the third ‘generation’, indicating a substantial contraction of the search space down to an optimal sub-space (Fig. 1b).

### Monitoring the convergence of parameter levels by population profiling

**Fig. 2.**
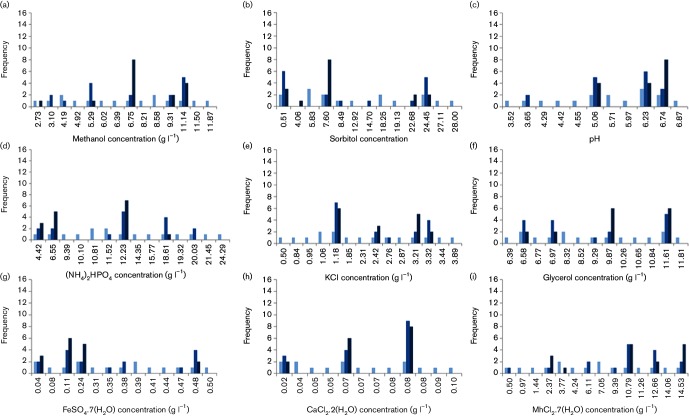
Population profiling for monitoring the absolute frequency of the levels of each factor tested in the population over generations. Absolute frequency denotes the number of times that level has been assigned to the ‘individuals’ in each ‘generation’. The factors represented here are: methanol (a), sorbitol (b), pH (c), (NH_4_)_2_PO_4_ (d), KCl (e), glycerol (f), FeSO_4_ . 7H_2_O (g), CaCl_2_ . 2H_2_O (h) and MgSO_4_ . 7H_2_O (i). The levels that appeared at least once in the search are displayed along the abscissa, and the absolute frequency is displayed along the ordinate. In each plot, the tone of the blue bars becomes darker for further ‘generations’ with the lightest shade representing the first ‘generation’ and the darkest tone representing the third, and last, ‘generation’ of the evolution experiments.

‘Population’ profiling is the investigation of how frequently each level is occupied by each factor through the course of ‘generations’. This approach allows the monitoring of the convergence of each factor towards a specific level, which guides the system to reach the optimum. Selection of a termination criterion to monitor the convergence in the output of the optimization study did not necessarily ensure the convergence of every factor towards an optimal value (Fig. 2). However, it suggested a refined multidimensional sub-space within which an optimum of the initial design space could lie. Exploiting this potential through a fine-tuning step improved the outcome of the optimization study even further. In our test case, the levels for methanol, sorbitol and pH showed a clear convergence towards unique values at 6.75, 7.60 and 6.74 g l^−1^, respectively (Fig. 2). In the case of ammonium, potassium and glycerol, the footprints in the third ‘generation’ indicated a pronounced convergence towards unique levels at 6.55, 3.21 and 9.87 g l^−1^, respectively. The optimal concentrations of FeSO_4_ . 7H_2_O and CaCl_2_ . 2H_2_O were determined based on the levels employed more frequently in the ‘population’ as the search approached the third generation. The optimal concentration of MgSO_4_ . 7H_2_O was determined by testing the two distinct levels that were more frequently adopted as the system evolved towards an optimum, and 2.37 g l^−1^ was identified to perform better (Supplementary Material). This strategy could also be adopted if one wished to terminate the labour-intensive GA prematurely, when an initial sign of convergence in the output was observed, and proceed to fine-tune the set-up through a curation stage, the latter would require decision-making on the levels to which the factors need to be assigned. It should also be noted that the GA approach does not ensure a global optimum. The search can lead us to a local optimum in the solution space and the identified optimum might change depending on the initial starting random ‘population’. From this perspective, the results should be evaluated in terms of the improvement achieved in the final objective.

We then challenged our optimized set of conditions against previously employed conditions. We compared our set’s performance with respect to minimal medium buffered at pH 6 or defined medium buffered at pH 5, both of which are commonly used by researchers in the field. The enzyme activity was shown to improve by up to 80 % when the optimized set of conditions was used instead (Supplementary Material).

### Exploration of an optimal sub-space via evolutionary algorithms

The data generated during the course of the conducted heuristic search employing the genetic algorithm were used to obtain a model to explain the relationship between the factors and the output response described by the scores of individual objectives. Linear regression models failed to describe the search space acceptably since a large number of factors and their interactions contributed to the construction of the search space under investigation. Although non-linear models provide a useful alternative in such instances, our lack of knowledge of the model structures opens up an unfeasibly large number of possibilities to be tested. Instead, we propose Symbolic Regression (SR) as an alternative approach to handle high-dimensional modelling problems with an unknown model structure. Symbolic Regression [24, 25] is a genetic programming approach, which starts with a random ‘population’ of individual models constructed from pre-defined allowable functions in a ‘plug-and-play’ fashion, and ‘evolves’ both the structure of the models and the coefficients of the model through the course of ‘generations’ until an acceptable fit is achieved.

In a standard DOE approach, since the number of the factors that are under investigation is kept relatively low in order to be practicable, multiple linear regression (MLR) can be employed to construct models that explain the variation in the response with a high goodness-of-fit. However, as the number of factors under investigation increases, more complex models are required to represent the possible interactions among those factors and to explain the variability in the output. For such applications, SR is a powerful tool since it does not require any *a priori* knowledge of the model structure or the provision of such information to the algorithm *ab initio*.

The size of the ‘population’, the number of ‘generations’, the number of factors that are allowed to have an interactive effect on the score in any individual model, and the ‘selection pressure’ for identifying better-fitting models to ‘mate’ and produce ‘offspring’ in the following ‘generation’ were previously reported as parameters that need case-specific adjustment in SR problems [24]. The size of the ‘population’ and the number of ‘generations’ are two parameters that need to be adjusted depending on the size of the dataset under investigation. For the current dataset of environmental conditions and the corresponding protein production yields, we tested different values for these parameters and observed that increasing both parameters up to 500 improved the search by identifying models that explained the variation in the data to a greater degree. Further increases in the size of the ‘population’ and/or the number of ‘generations’ did not yield further improvements in the performance of the models (Supplementary Material). Indeed, the selected parameter values were previously shown to be sufficient to analyse much larger biological datasets [25]. Only basic mathematical operations (addition, subtraction, multiplication and division) were allowed in the models to ensure the development of relatively simple model structures.

Next, we constructed four batches of 10 models using the same dataset, each model representing a unique course of ‘evolution’ and each batch representing how the system was used to describe our different objectives: high final cell density during the growth-promoting phase; little further growth during the protein production phase; high enzyme activity; and high specific productivity. These models allowed us to explain the variation in each individual objective caused by the nine factors under investigation (Supplementary Material). The residual sum of squares (R^2^) was selected as the metric to represent the proportion of variance in the models. The performance of these models in representing the variance in the dataset was compared to that of the MLR models, which are commonly employed in the standard DOE strategy (Table 2). SR outperformed MLR in explaining the variance of the dependent variables, i.e. the individual objectives in all four batches of 10 models. The highest goodness-of-fit (R^2^-value) for each batch is given in Table 2.

**Table 2.** Evaluation of the SR models for each individual objective

		R^2^	Adjusted R^2^*
OD_bi_	SR	0.645	0.583
	MLR	0.131	−0.021
OD_(ai−bi)_	SR	0.745	0.685
	MLR	0.332	0.173
Enzyme activity (Ea)	SR	0.804	0.758
	MLR	0.595	0.499
Specific productivity (P)	SR	0.881	0.853
	MLR	0.610	0.518

*R^2^ that has been adjusted for the number of predictors in the model.

We conducted sensitivity analyses employing these batches of 10 models to determine the sensitivity of each individual objective to a small variation in each factor. We shifted the value of each factor from its determined optimum by 10 % and investigated whether a similar response of 10 % or higher was observed in individual objectives in their respective model pools (Supplementary Material). If such a response was observed in the batch of models for each individual objective, we denoted this factor/predictor as a major contributor (Table 3). The sensitivity analysis revealed the distinction between what we call ‘operation-related’ factors and ‘cell culture-related’ factors in the experimental design, although the process of model construction was blind to the nature of the factors under investigation. We identified all dependent variables in the objective function to be highly sensitive to variations in the pH of the working culture. In this way, it was found to be imperative to have strict control over the pH of the cultivation during HuLy production by *K. phaffii* under the control of the alcohol oxidase promoter. The experiments showing total loss of protein activity under conditions where the pH was not controlled throughout the course of the fermentation (Supplementary Material) also confirm this model prediction.

**Table 3.** Summary of major contributor factors for each individual objective

	OD_bi_	OD_(ai−bi)_	Ea	P
pH	**√**	**√**	**√**	**√**
Glycerol		**√**	**√**	**√**
Ammonium		**√**	**√**	
Methanol	**n**/a*		**√**	**√**
Sorbitol	n/a*			**√**
Calcium				**√**
Potassium				
Iron				
Magnesium				

*n/a, not applicable since cultivation medium did not contain methanol and sorbitol during the pre-induction phase.

### CamOptimus: compiled version of the tool and the standalone GUI

We have proposed here an AI-based approach to tackle the complex multi-parameter experimental design problems encountered by biologists on a day-to-day basis. Ease-of-use, accessibility and flexibility are features that help any tool to be adopted by a broad community of researchers. Therefore, we have developed a compiled version of this tool and made it available as a stand-alone GUI, which only requires the freely available MATLAB Runtime Environment to be installed. The GUI has a modular structure, which guides the user through the stages of designing a problem and optimizing the accompanying experimental set-up. The interface allows the user to proceed to the next steps in the process only after the prerequisite information has been provided, and this helps to define the structure of the experiments that need to be carried out.

**Fig. 3.**
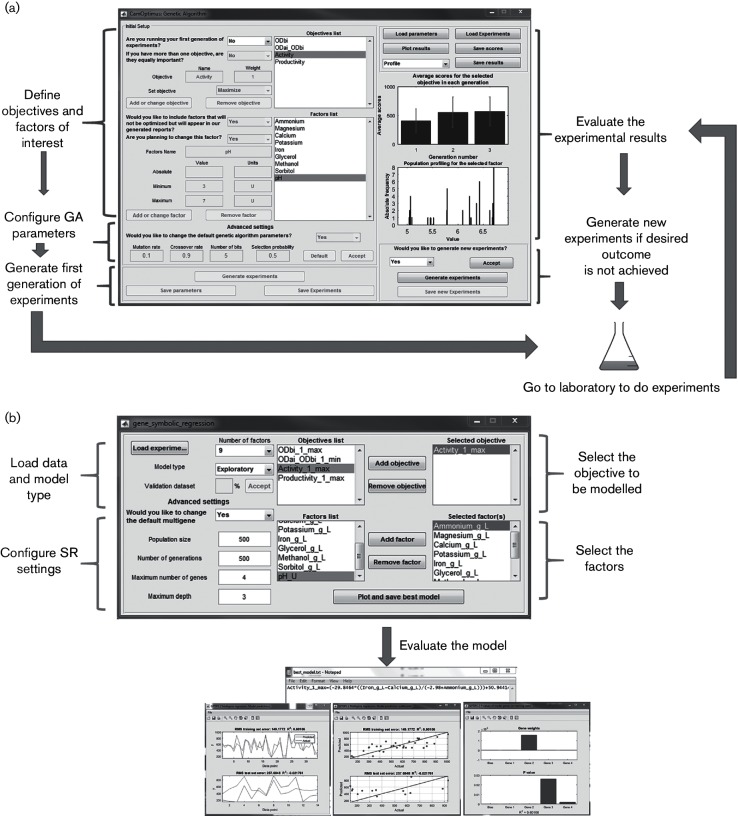
Example input/output entries for the CamOptimus GUI and the process algorithm. A test system investigating nine parameters for optimizing four objectives is provided above. The optimization search is conducted for three generations using the GA (a) and the data generated are modelled by SR (b). The algorithms for conducting the optimization search and analyzing the data are shown as annotations to the GUI screenshots.

The user first provides the biological objectives and the factors relevant to the experimental design, along with their allowable ranges. The default parameters of the GA are modified, if necessary, at this initial stage. Once this configuration is established, the user is no longer allowed to alter any one of these items throughout the course of the optimization procedure, in order to prevent any possible errors. The user is then prompted to create a set of experiments, carry out these experiments in the laboratory and measure the outcomes, provide these results back to the tool to allow it to calculate the ‘fitness’ and evaluate the comparative information on the convergence of the scores in a repeated manner until a satisfactory outcome, as decided by the user, is achieved. The user may also proceed to investigate the level-profiles of each parameter following its course in all ‘generations’, in case fine-tuning of the results may be needed in the population-profiling component. This procedure marks the end of the GA component of the tool (Fig. 3a). The second, and last, component of the tool, which employs SR, allows the user to investigate the possible-solution space to conduct a sensitivity analysis on their individual objectives to determine which of the factors had the greatest influence on the optimization exercise (Fig. 3b). The video S1 (available in the online Supplementary Material; CamOptimus_demo.mov), shows how the tool is used.

In summary, we have proposed a hybrid approach, which employs evolutionary algorithms, to address the multi-parametric experimental design problem. We have presented CamOptimus, a simple-to-use and freely available tool, which adopts the ‘best of both worlds’ from the DOE and GA methodologies. The possible solution space is investigated through GA, which will most likely ensure that the search leads to the discovery of the optimum sub-space within the confines of the initial search-space, without imposing artificial restrictions on either the number of factors that need to be investigated or their levels. The algorithm guides the search towards a more confined space, where an optimum solution resides, depending on how different sets of conditions perform in achieving the desired objective and thus allows the identification of optimal space without testing all possible combinations. A regression-based analysis is then employed to construct models using the wealth of experimental data generated within the search-space in order to analyse the sensitivity of the system to individual factors. We tested this platform on a case study of biotechnological significance, within the domain of recombinant protein production by microbial hosts, and the levels of nine interdependent environmental cultivation parameters were optimized in three generations by testing only 16 different conditions in each generation. The optimum levels of factors were determined by monitoring distribution of the frequency of the level occupation over successive generations. Comparison of the optimized condition with ones previously reported in the literature revealed that up to 80 % improvement in protein production was achieved under optimized conditions. The outstanding success of the new experimental set-up in comparison to those available in the literature demonstrates the possible scope for such an optimization exercise in improving the efficiency of many other commonly employed methods that might potentially be operating under sub-optimal conditions. In fact, the tool has been successfully employed in a completely different domain; in optimizing environmental conditions for enhancing macropinocytosis by *Dictyostelium* amoebae by the Kay lab (MRC Laboratory of Molecular Biology, UK), thus demonstrating its flexibility and adaptability for different experimental problems (unpublished work). We believe CamOptimus to be an attractive (and free) alternative to commercially available DOE software for use in both academic and industrial applications.

## Supplementary Data

882 Supplementary File 1

## Supplementary Data

7134 Supplementary File 2

## Supplementary Data

90 Supplementary File 3
