# Supplementary material for: CamOptimus: a tool for exploiting complex adaptive evolution to optimize experiments and processes in biotechnology
Source: Microbiology (Reading). 2017 Jun 21;163(6):829–39. doi: 10.1099/mic.0.000477 (PMC5817226; doi:10.1099/mic.0.000477)
Supplement: Supplementary File 1 [file mic-163-829-s001.pdf]

CamOptimus: A Tool for Exploiting Complex Adaptive Evolution to Optimise Experiments and Processes in Biotechnology

Ayca Cankorur-Cetinkaya, Joao M L Dias, Jana Kludas, Nigel K.H. Slater,Juho Rousu, Stephen G. Oliver*, Duygu Dikicioglu

Supplementary Material

Text S1. Supplementary information on test case

Data S1. Raw data for GA and 4 batches of 10 models in SR for each individual objective

This spreadsheet is comprised of 4 tabs each dedicated to the batch of 10 "evolutions" achieved for a specific objective of the combined objective function; final cell density during the growth-promoted phase (ODbi tab), further growth during the protein production phase (ODai-ODbi tab), enzyme activity (Ea tab), and specific productivity (SP tab). The models and the cognate R^2 values are provided along with the individual compositions in the "population" in 3 "generations" and the experimental values measured for each individual objective.

Movie S1. Demo video for CamOptimus

Text S1

Determination of the biological objectives for recombinant protein production under inducible promoters

The expression system employed made use of a strong methanol-inducible promoter to promote recombinant protein production and suggested that the fermentation should be divided into two main phases. The culture should first undergo a biomass-generation phase, in which the maximum possible cell density would be attained for use in the second, product-generation phase. In this second phase, metabolic flux should be directed away from cell

growth and towards the production of the recombinant protein once its synthesis had been induced. This fermentation regime suggested that four main biological objectives should be selected for our system: (i) maximising cell density prior to induction of recombinant protein synthesis, (ii) maximising the biological activity of the recombinant enzyme at the end of the fermentation, (iii) maximising the culture's specific productivity, and (iv) minimising any further increase in cell density during the induction phase.

Selection of factors and their boundaries to be employed in GA

Published concentrations of medium components that acted as the major nitrogen, potassium, magnesium, and calcium sources in *K. phaffii* varied substantially in studies employing different clones or different growth strategies [1–4]. Among the micronutrients, iron displayed the largest variation in concentration between different defined medium recipes [4,5]. Cells that employ the *AOX* promoter to express the recombinant protein have usually been cultivated on sorbitol or glycerol prior to induction, with a co-feeding strategy based on methanol/sorbitol mixtures employed during the induction period [3, 6]. Preliminary shake-flask experiments revealed that HuLy activity was lost from batch cultures whose pH was not controlled throughout the course of the fermentation, and so pH was included as another factor to be optimised.

The maximal and the minimal concentrations of medium components previously reported in the literature were used to set the allowable ranges for each factor [4,5,7–9] Although pH values between 5 and 6 have been used in most studies, *K. phaffii* can grow in a wide range pH from 3 to 7 [10]. The pH of the culture can affect proteolytic activity, secretion, and protein production [11]; therefore, we kept the pH range as wide as possible in order to identify the optimum value for HuLy production.

Investigation of the effect of the citrate/phosphate buffer on culture performance

The effect of the addition of buffer components to maintain constant pH on the growth and production characteristics of the culture was investigated at different stages of the analysis. Preliminary analysis involved growing the microbial culture in shake flasks, where the culture pH was maintained constant in buffered conditions, or was allowed to decrease in non-buffered defined medium. The protein activity diminished in cultures where the pH was not kept constant, thus demonstrating the necessity for pH control. For the operating conditions investigated in this study, the working pH was in the range of 3-7. Citrate/phosphate buffer, with reported functionality in the pH range of 2.6-7 [12], was selected as a suitable option for our purposes, since its constituents did not pose a threat of toxicity for the culture. Since citric acid and dibasic sodium phosphate were used to prepare the buffer, possible interference due to buffer components acting as macronutrients was investigated to rule out such complications. Varying the concentration of citric acid (5-fold) to account for the possible variation it would display in the pH range of 2.6 – 7 (<5-fold) did not yield a change in growth or protein activity. In order to investigate the effect of the phosphate group acting as a macronutrient, a similar experiment was carried out as described for testing the effect of the citrate component of the buffer. Furthermore, in order to test the phosphate group specificity of the analysis, dibasic sodium phosphate was replaced by sodium sulphate, to provide equivalent Na molarities. The difference in growth or protein activity always remained within 10% of one another and that of the reference study, regardless of the concentrations employed within the limits that were of interest for the purpose of this study (Table A). Therefore the citrate/phosphate buffer was considered as an acceptable option for the cultivations performed in snap-cap tubes. Throughout the evolutionary procedure, the pH of randomly selected cultures was measured in order to ensure that the buffer was working satisfactorily. The complete data collected over three

generations were investigated for the possible existence of any correlation between any single objective and the buffer components individually and no trends were detected with all regression coefficients (R^2) remaining <0.10 (Figure A). We then extended our investigation of the citrate component further by tracing any possible indication that its chelating properties impacted on the availability of calcium or iron, both of which were under consideration within the scope of this work. Despite earlier reports on the calcium- and iron-chelating properties of citric acid [13], the concentrations employed in the study were shown not to cause such an effect throughout the course of the study, with the Pearson correlation coefficients for both cases remaining below 0.3 and the regression coefficients below 0.1 (Figure B).

Table A. Effect of buffer components on growth and protein production.

	**% change in OD**	**% change in HuLy activity**
**Citric acid supplementation**	2.362	8.456
**NaH_2PO_4 supplementation**	5.133	7.730
**Na_2SO_4 supplementation**	7.605	4.351

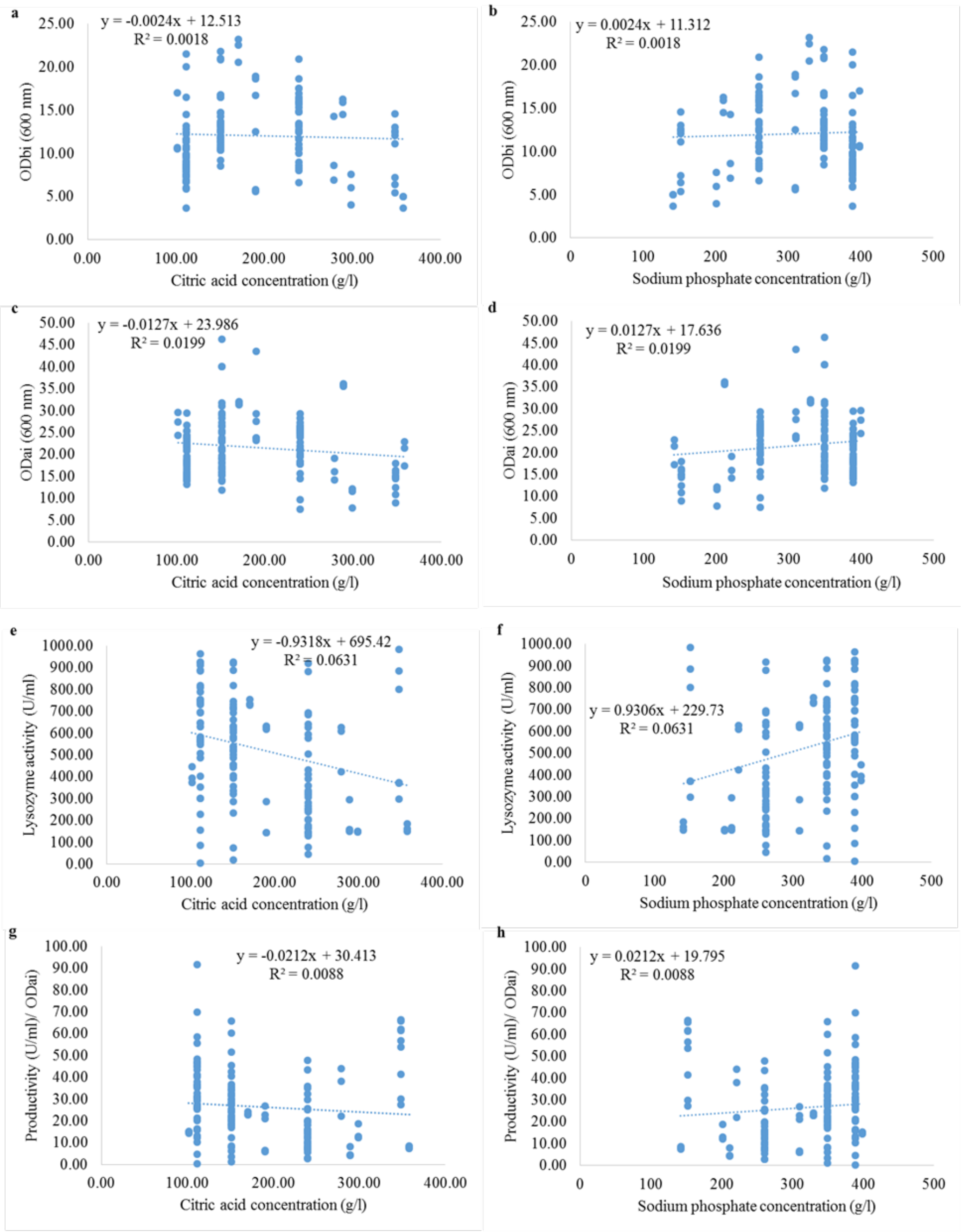

Figure A. Investigation of the possible correlation between individual objectives and the concentration of the buffer components.

Complete data set comprised of all measured objectives over three generations. The changes in biomass during the growth promoted phase (OD_{bi}) as a function of citric acid concentration (a) or dibasic sodium phosphate concentration (b), the final biomass attained (OD_{ai}) as a function of citric acid concentration (c) or dibasic sodium phosphate concentration (d), the HuLy activity as a function of citric acid concentration (e) or dibasic sodium phosphate

concentration (f), and the culture productivity as a function of citric acid concentration (g) or dibasic sodium phosphate concentration (h) are provided along with their linear regression models and R^2 is provided as a goodness-of-fit measure.

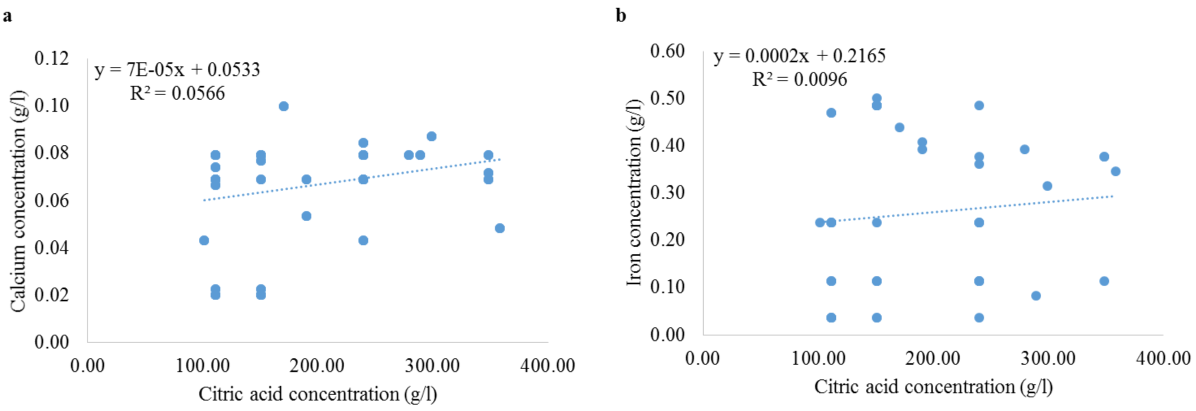

Figure B. Investigation of the possible chelation effect of citric acid on calcium and iron in the cultivation medium.

Distribution of calcium (a) and iron (b) concentrations tested over three generations as a function of the citric acid concentrations of the corresponding sample. Any possible trends and correlations were investigated by fitting linear models (R^2 as a goodness-of-fit measure). The slopes of the models indicating the existence of possible trends were negligibly small.

Fine-tuning the global optimum by population profiling

We carried out fine-tuning of the optimised environmental conditions that we had obtained at the end of the 3rd "generation" in the GA study by employing a population profiling methodology to determine whether the performance of the system could be improved even further. We investigated the change in how many individuals assumed each level over the 3 "generations" for each one of the 9 factors (Figure 2). We calculated the percent occupancy of the levels in the better-performing, i.e. the "fitter" fraction of the "population" in the last "generation", which corresponded to the half of the "population" with the highest "fitness"

scores, and employed this as a footprint for identifying the optimal levels for each factor (Figure C). The footprints of methanol, sorbitol and pH in the 3rd "generation", as well as their convergence profiles through the course of the GA search, indicated that the levels that these factors assumed converged towards unique values at 6.75g/L, 7.60g/L and 6.74, respectively (Figure 2(a-c) and Figure C(a-c)). In the case of ammonium, potassium and glycerol, although the convergence profiles displayed bi-modal behaviour, the footprints in the 3rd "generation" indicated a pronounced convergence towards unique levels at 6.55 g/L, 3.21 g/L and 9.87 g/L, respectively (Figure 2(d-f) and Figure C(d-f)). The convergence of the footprint distribution for the remaining 3 factors either were not as pronounced, e.g. iron (Figure C(g)), or displayed bi-modal behaviour, e.g. calcium and magnesium (Figure C(h-i)). The optimal concentration of FeSO4.7(H_2O) was determined as 0.04 g/L. The distribution of the frequency of the level occupation over successive generations was investigated for the factors, whose footprints displayed bi-modal distributions. The level of 0.07g/L for $CaCl_2$.2(H_2O) was employed more frequently in the "population" as the search approached the 3rd generation (Figure C(h)) and therefore this level was selected as the optimal concentration for $CaCl_2$.2(H_2O). A similar inspection of the progression of the population profiles for $MgSO_4$.7(H_2O) indicated that two distinct levels were more frequently adopted as the system evolved towards an optimum (Figure C(i)). Therefore, both levels were tested experimentally to identify the better performing condition. The lower of these two levels improved the activity of the secreted HuLy enzyme and the specific productivity by nearly 20- and 15-fold, respectively. The adoption of the lower value also resolved issues related to the medium precipitating, thus substantially improving reproducibility. The $MgSO_4 7{\cdot}H_2O$ level for the optimal "individual" was selected as 2.37g/L.

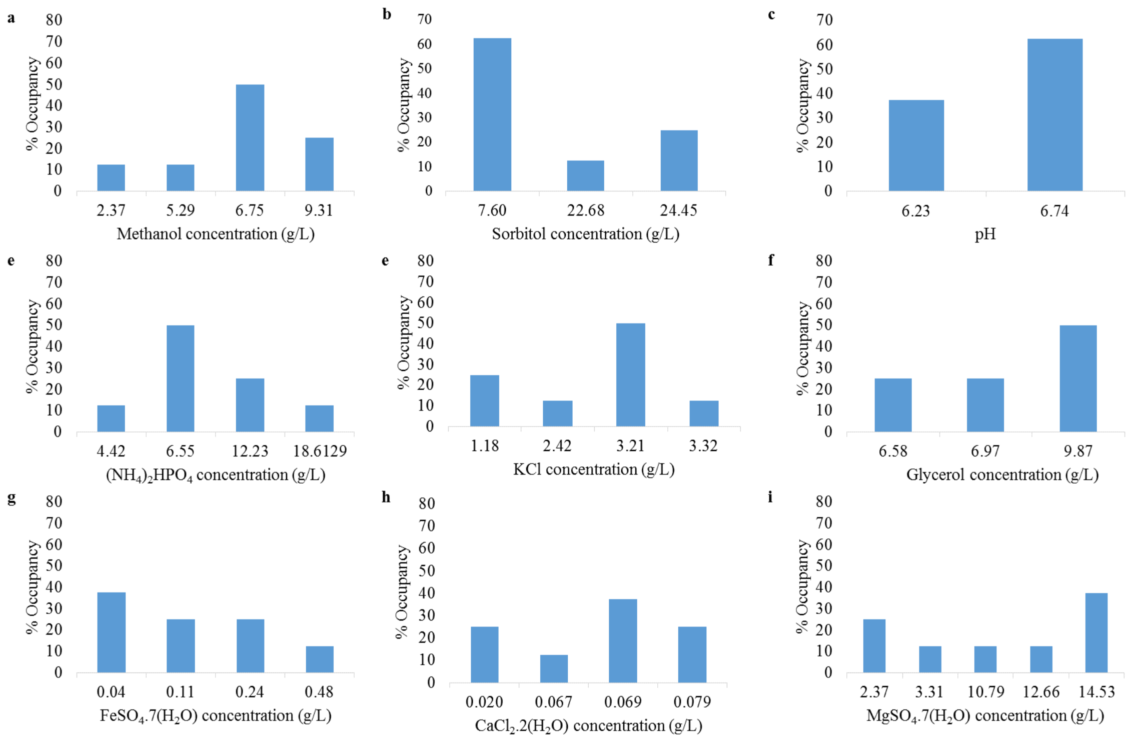

Figure C. Relative occupancy of the levels of each factor in the better performing fraction of the 3rd generation

Relative occupancy of the levels assigned in the better performing fraction are displayed (i.e. the 50% of the population with the highest "fitness" scores, of the 3rd generation for each factor). Methanol (a), sorbitol (b), pH (c), $(NH_4)_2PO_4$ (d), KCl (e), glycerol (f), $FeSO_4.7(H_2O)$ (g), $CaCl_2.2(H_2O)$ (h), and $MgSO_4.7(H_2O)$ (i). The footprint was generated by plotting the relative distribution of the instances a given level was employed in the better-performing half of the 3rd generation. The levels, which appeared at least once in the last generation, is displayed on the abscissa and the % occupancy, indicating the relative representation of each level, is displayed in the ordinate.

Challenging the optimised cultivation conditions against the existing conditions

Buffered minimal medium, containing yeast nitrogen base, and defined medium [4,14] were frequently reported as the cultivation environment for *K. phaffi* strains expressing

recombinant proteins under the control of the AOX promoter. pH values between 5 and 6 are used in most studies involving *K. phaffii* fermentations [10]. We challenged the optimised environmental conditions against these commonly employed conditions to assess the performance of the methodology. We showed that the activity of the secreted enzyme in the culture could be increased by 30% and 80% through adopting the optimised conditions to replace minimal medium buffered at pH 6 and defined medium buffered at pH 5, respectively (Figure D).

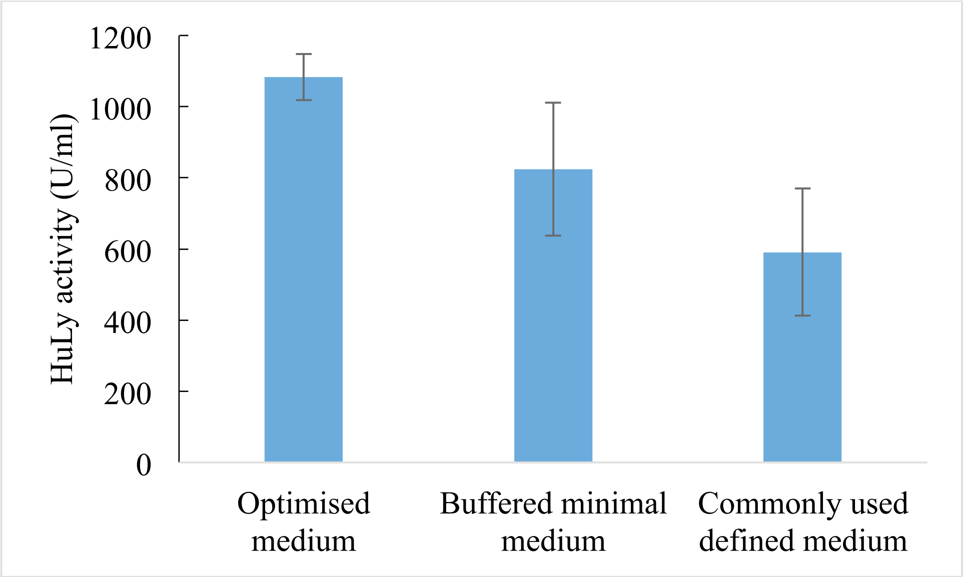

Figure D. Challenging the performance of *K. phaffii* cultivated under optimised conditions.

The error bars represent the variation between the technical triplicates. The variation in the cultures investigated under the optimised conditions was the lowest among the three different cultivation conditions that were tested.

Determination of Population Size and Number of Generations for SR Analyses

In order to identify the suitable parameter setting, regression analyses were conducted for a number of combinations of population size and generation number to model the protein

activity (Table B). The goodness of fit values were provided as an average of 3 independent simulations for each setting.

Table B. Effect of Population Size and Number of Generations on Goodness of Fit

Population Size	Number of Generations	Goodness of Fit of the Model
100	100	0.772±0.022
100	500	0.799±0.007
500	100	0.811±0.018
500	500	0.814±0.012
500	1000	0.795±0.025
1000	500	0.796±0.032
1000	1000	0.810±0.014

Sensitivity of each objective to each factor as determined by SR models

Growth yield during the protein production phase was determined to be highly sensitive to changes in the concentrations of glycerol and ammonium in the cultivation environment. This was an expected outcome since both the preferred carbon source (glycerol) and the preferred nitrogen source (ammonium) become limiting during that phase in the fed-batch culture leading to incremental changes that are readily reflected in the growth yield. Enzyme activity was also highly sensitive to the variations in the availability of glycerol and ammonium since the production of the recombinant protein necessitated competing with growth for the available resources. In addition, enzyme activity was also highly sensitive to variations in methanol concentration, which was also expected since the production system was activated by methanol inducing the alcohol oxidase promoter. Specific productivity, a compound objective, was highly sensitive to variations in all potential carbon sources; both available

(methanol and sorbitol), and unavailable (glycerol) during the protein production phase. Additionally, we identified specific productivity to be highly sensitive to variations in the concentration of calcium. This was an unexpected outcome since Human Lysozyme has been reported not to bind calcium [15]. Moreover, calcium was reported to merely contribute to the stability of calcium-binding lysozymes [16]. The results highlighted here may suggest the existence of a possible universal role for calcium for the lysozyme family, which requires further investigation. The models constructed in this study were not able to capture the variance in the objectives that was caused by potassium, iron, or magnesium since none of the objectives were identified to be sensitive to variations in either one of these predictors. Specifically, magnesium was not included as a predictor in many of the constructed models (Figure E) and the bi-modal distribution of its levels in the search-space of the GA was thought to contribute to that effect.

		model 1	model 2	model 3	model 4	model 5	model 6	model 7	model 8	model 9	model 10
**a** **OD bi**	Ammonium	0.89	0.29	1.28	0.85	0.00	1.53	1.64	0.32	1.16	0.09
	Potassium	1.56	0.66	3.10	2.45	2.98	2.10	0.43	2.70	0.16	5.61
	Magnesium	1.04	0.14	1.01	1.89	0.17	0.51	0.26	0.84	N/A	0.05
	Iron	3.15	0.05	0.01	0.04	2.77	4.25	2.54	0.08	1.61	0.06
	Calcium	1.89	1.02	1.66	1.77	2.07	2.90	N/A	0.08	0.57	1.69
	Glycerol	0.03	3.56	1.82	0.40	9.85	6.14	N/A	0.95	9.57	0.20
	pH	31.30	61.03	98.32	29.67	23.10	48.18	52.06	12.71	96.50	94.58
**b** **OD ai-bi**	Ammonium	6.37	12.62	N/A	0.19	0.57	0.45	N/A	0.03	1.45	1.77
	Potassium	1.54	0.64	0.95	0.07	4.56	0.45	3.84	0.19	1.68	3.98
	Magnesium	0.02	0.38	0.99	1.71	0.15	2.08	0.47	0.55	0.06	1.36
	Iron	3.19	0.15	0.13	0.04	3.94	0.03	0.24	0.13	0.26	0.55
	Calcium	N/A	0.66	1.77	1.42	0.80	3.04	4.06	0.43	4.54	2.15
	Glycerol	1.69	41.97	8.72	5.28	4.32	0.70	15.27	6.08	15.00	21.16
	Methanol	3.71	2.13	0.11	2.61	3.10	4.87	N/A	0.08	N/A	N/A
	Sorbitol	0.11	1.43	0.56	3.40	1.12	3.30	0.06	1.66	0.66	3.97
	pH	0.49	15.67	14.58	0.08	5.01	5.34	0.92	2.19	5.08	11.13
**c** **Enzyme activity**	Ammonium	1.06	0.96	5.68	16.19	4.65	0.20	1.30	0.20	1.48	1.07
	Potassium	0.70	0.06	2.81	0.76	1.27	0.98	0.76	0.95	0.87	0.51
	Magnesium	N/A	N/A	0.33	N/A	0.30	0.19	0.10	0.09	N/A	0.65
	Iron	2.65	0.04	0.08	2.40	1.58	3.89	1.01	2.19	4.00	1.47
	Calcium	3.63	0.78	0.93	2.64	7.07	0.38	2.58	2.14	0.96	0.16
	Glycerol	9.63	10.77	8.22	0.92	6.96	18.13	11.97	8.80	20.90	5.63
	Methanol	29.24	N/A	5.22	3.14	2.97	9.98	0.31	0.13	13.17	4.39
	Sorbitol	1.05	2.90	1.17	1.10	0.14	6.20	1.35	0.47	1.33	0.02
	pH	28.12	49.58	6.77	6.75	11.33	24.25	8.17	29.43	26.87	2.78
**d** **Specific productivity**	Ammonium	5.11	1.28	0.95	1.58	3.96	1.21	0.66	5.79	0.99	2.16
	Potassium	1.87	4.51	0.29	1.70	0.41	1.63	0.01	0.20	4.22	0.13
	Magnesium	0.16	N/A	N/A	N/A	N/A	N/A	0.02	0.55	0.08	0.10
	Iron	0.01	0.15	3.01	2.76	2.31	0.37	0.57	0.05	4.20	3.44
	Calcium	N/A	0.25	1.39	0.03	0.67	0.20	N/A	0.30	10.61	1.26
	Glycerol	7.12	17.22	9.72	3.13	2.59	11.69	4.19	6.35	22.04	0.09
	Methanol	2.73	7.99	35.51	3.55	3.07	2.94	61.94	60.90	5.34	7.63
	Sorbitol	85.25	2.02	2.79	3.45	0.15	2.29	0.01	4.82	23.42	2.99
	pH	3.69	11.66	29.83	2.70	8.53	8.37	60.81	64.46	7.79	N/A

Figure E. Heat map of the sensitivity of each individual objective to variations in each individual factor in the model pools

The sensitivity of each objective [final cell density during the growth-promoted phase (ODbi) (a), further growth during the protein production phase (ODai-ODbi) (b), enzyme activity (Ea) (c) and specific productivity (SP) (d)] to a variation in the value of each factor of ±10%. The shade of green denotes the strength of the response, the corresponding values numerically denote its strength, in percent terms, for each model.

References

1. **Cos O, Ramón R, Montesinos JL, Valero F**. *Operational strategies, monitoring and control of heterologous protein production in the methylotrophic yeast Pichia pastoris*

under different promoters: a review. Microb Cell Fact. 2006;5:17.

2. **Gach JS, Maurer M, Hahn R, Gasser B, Mattanovich D, et al.** *High level expression of a promising anti-idiotypic antibody fragment vaccine against HIV-1 in Pichia pastoris*. *J Biotechnol*. 2007;128(4):735–46.

3. **Hesketh AR, Castrillo JI, Sawyer T, Archer DB, Oliver SG**. *Investigating the physiological response of Pichia (Komagataella) pastoris GS115 to the heterologous expression of misfolded proteins using chemostat cultures*. *Appl Microbiol Biotechnol*. 2013;97(22):9747–62.

4. **Prielhofer R, Maurer M, Klein J, Wenger J, Kiziak C, et al.** *Induction without methanol: novel regulated promoters enable high-level expression in Pichia pastoris*. *Microb Cell Fact*. 2013;12(1):5.

5. **Delic M, Mattanovich D, Gasser B**. *Monitoring intracellular redox conditions in the endoplasmic reticulum of living yeasts*. *FEMS Microbiol Lett*. 2010;306(1):61–6.

6. **Niu H, Jost L, Pirlot N, Sassi H, Daukandt M, et al.** *A quantitative study of methanol/sorbitol co-feeding process of a Pichia pastoris Mut+/pAOX1-lacZ strain*. *Microb Cell Fact*. 2013;12(1):33.

7. **Baumann K, Maurer M, Dragosits M, Cos O, Ferrer P, Mattanovich D**. *Hypoxic fed-batch cultivation of Pichia pastoris increases specific and volumetric productivity of recombinant proteins*. *Biotechnol Bioeng*. 2008;100(1):177–83.

8. **Pfeffer M, Maurer M, Stadlmann J, Grass J, Delic M, et al.** *Intracellular interactome of secreted antibody Fab fragment in Pichia pastoris reveals its routes of secretion and degradation*. *Appl Microbiol Biotechnol*. 2012;93(6):2503–12.

9. **Çelik E, Çalik P, Oliver SG**. *Fed-batch methanol feeding strategy for recombinant protein production by Pichia pastoris in the presence of co-substrate sorbitol*. *Yeast*.

2009;

10. **Cregg JM, Vedvick TS, Raschke WC**. *Recent Advances in the Expression of Foreign Genes in Pichia pastoris*. *Bio/Technology*. 1993;11(8):905–10.

11. **Fakruddin M, Mazumdar MR, Mannan BKS, Chowdhury A, Hossain MN**. *Critical Factors Affecting the Success of Cloning, Expression, and Mass Production of Enzymes by Recombinant E. coli*. *ISRN Biotechnol*. 2013;(3):1–7.

12. **Pearse A**. *Histochemistry: Theoretical and applied. Am J Med Sci*. 1980;97–158.

13. **Ash M, Ash I**. *Handbook of Green Chemicals*. *Synapse Info Resources*; 2004. 1348 p.

14. **Kumita JR, Johnson RJK, Alcocer MJC, Dumoulin M, Holmqvist F, et al.** *Impact of the native-state stability of human lysozyme variants on protein secretion by Pichia pastoris*. *FEBS J*. 2006;273:711–20.

15. **Kissinger CR, Parge HE, Knighton DR, Lewis CT, Pelletier LA, et al.** *Crystal structures of human calcineurin and the human FKBP12-FK506-calcineurin complex. Nature*. 1995;378(6557):641–4.

16. **Vogel HJ**. *Calcium-Binding Protein Protocols*. Vol. 172. *Humana Press*; 2002.
